# Parálisis Flácida Aguda y Encefalitis, una Combinación Mortal. Reporte del Último Caso de Rabia Humana en Colombia: Recordatorio de un Enemigo Prevenible

**DOI:** 10.31083/RN49292

**Published:** 2026-03-25

**Authors:** Francisco Aureliano García Jimenez, Paula Andrea Valencia-Rey, Kevin Tutalchá Oviedo, Santiago Mejía López, Juan Carlos Arango Viana, Juan David Cuartas Ramírez

**Affiliations:** ^1^Departament of Antioquia, Fundación Hospitalaria San Vicente de Paul, 050010 Medellin, Colombia; ^2^Departament of Antioquia, Universidad de Antioquia, 050010 Medellin, Colombia

**Keywords:** rabia humana, Colombia, encefalitis aguda, parálisis flácida aguda, zoonosis, encefalitis viral, human rabies, Colombia, acute encephalitis, acute flaccid paralysis, zoonosis, viral encephalitis

## Abstract

**Introducción:**

En países con baja incidencia de rabia humana, este virus letal rara vez se considera en el diagnóstico diferencial de la parálisis flácida aguda.

**Caso Clínico:**

Mujer joven con fiebre y debilidad progresiva que evolucionó a cuadriparesia flácida, asociada a síntomas neuropsiquiátricos, neuropatía craneal múltiple y disautonomía. Se exponen las pruebas complementarias y los peculiares reflejos que acompañaron el diagnóstico de muerte encefálica. El diagnóstico se confirmó post mortem mediante hallazgos histopatológicos compatibles.

**Conclusiones:**

La rabia humana debe considerarse en cuadros de parálisis flácida con encefalitis debido a su implicación en la prevención de otras muertes mediante medidas de salud pública.

## Abstract (en)

**Introduction:**

In countries with a low incidence of human rabies, this lethal virus is rarely considered in the differential diagnosis of acute flaccid paralysis.

**Clinical Case:**

A young woman presented with fever and progressive weakness that evolved into flaccid quadriparesis, associated with neuropsychiatric symptoms, multiple cranial neuropathy, and dysautonomia. The complementary tests and the unusual reflexes that accompanied the diagnosis of brain death are described. The diagnosis was confirmed postmortem by compatible histopathological findings.

**Conclusions:**

Human rabies should be considered in cases of flaccid paralysis with encephalitis due to its implications for preventing further deaths through public health measures.

## 1. Introducción

En países con baja incidencia de rabia humana, esta entidad suele omitirse en el diagnóstico diferencial de la parálisis flácida aguda. Presentamos un caso de rabia humana con manifestaciones neurológicas y neuropsiquiátricas que evolucionó rápidamente a muerte encefálica. Se describen los hallazgos clínicos, paraclínicos y neuropatológicos que permitieron confirmar el diagnóstico, resaltando su importancia como causa infrecuente pero letal de compromiso neurológico agudo. Este reporte de caso fue elaborado siguiendo las guías CARE (CAse REport), y la lista de verificación CARE se proporciona como **Material Suplementario**.

## 2. Presentación del Caso

Femenina de 24 años, sin antecedentes patológicos. Consulta por cuadro clínico de una semana de dolor en el brazo derecho no asociado a evento traumático, acompañado de fiebre y debilidad muscular progresiva. Inicialmente se comportó como una monoparesia del miembro superior derecho, con progresión a diparesia braquial y, posteriormente a cuadriparesia, la cual llevó a la postración, tres días después del inicio de síntomas. Adicionalmente, se hicieron manifiestos síntomas neuropsiquiátricos como agitación psicomotora, delirios de persecución y erotomaníacos, alucinaciones visuales, desorientación temporo-espacial y lenguaje incoherente. A los 7 días de evolución, se sumó disfagia y pérdida del control de esfínteres por lo que deciden consultar a un hospital de alta complejidad en Medellín, Colombia.

Al examen físico se documentó fiebre hasta 38,6 °C. Se encontró epífora, diplopía, disfagia, ptosis palpebral bilateral e inyección conjuntival, explicado por compromiso de los nervios craneanos oculomotores, IX y X. Además, presentaba cuadriparesia asimétrica, de predominio distal, acompañada de disminución de los reflejos miotendinosos con predominio en extremidades inferiores y extremidad superior derecha. No tenía excoriaciones ni mordeduras en la piel. Ese mismo día, debutó con somnolencia, sialorrea e insuficiencia respiratoria por lo que requirió intubación y ventilación mecánica. Progresó con taquicardia persistente y labilidad de la presión arterial secundario al compromiso autonómico.

Se trató de un síndrome de debilidad generalizada en presencia de cambios neurocognitivos que llevaron a enfocar el caso como encefalitis aguda, incluyendo posibles enfermedades autoinmunes e infecciosas. Se realizó una punción lumbar con presión de apertura de 30 cmH_2_O, citoquímico de líquido cefalorraquídeo (LCR) con proteínas 98,7 mg/dL, glucosa 59 mg/dL, 10 leucocitos por mm^3^, tinción de gram negativa, baciloscopia y tinta china negativas. Se descartó infección por virus herpes simple 1 y 2 (HSV-1/2), virus inmunodeficiencia humana (VIH), leptospirosis, sífilis, complicaciones postinfecciosas de influenza A o coronavirus 2 del síndrome respiratorio agudo grave (SARS-CoV2). Reacción en Cadena de la Polimerasa (PCR) específica para herpes y panel para meningitis BIOFIRE® FILMARRAY® (BioMérieux) fue negativo. Se descartó también tuberculosis meníngea y criptococosis.

Su acompañante mencionó contacto con animales domésticos y de granja en su vivienda, además de la muerte de un gato un mes previo al inicio de los síntomas y algunas aves de corral sin causa aparente; esto obligó a pensar en zoonosis incluyendo entre otras, influenza aviar y virus de la rabia. Dado el rápido deterioro neurológico se trató inicialmente con aciclovir, ceftriaxona y doxiciclina, con el objetivo de tratar causas como Virus Herpes Simple, *Leptospira interrogans* y Rickettsias.

Se realizó una tomografía simple de cráneo al ingreso que no evidenció lesión estructural. La inestabilidad hemodinámica limitó el estudio con resonancia cerebral. La electromiografía al ingreso reportó disminución atípica de las amplitudes de los potenciales de acción motores tanto a nivel proximal como distal, sin daño axonal activo, sin compromiso sensitivo ni hallazgos de enfermedad desmielinizante. Se solicitaron estudios de autoinmunidad en los cuales resultaron anticuerpos antinucleares positivos 160 diluciones con patrón granular fino, por lo que también recibió 1 dosis de inmunoglobulina humana. A los 3 días de su ingreso, presentó un evento convulsivo tónico clónico generalizado que obligó a llevar a sedación profunda y adicionar terapia anticrisis.

Se realizaron tres electroencefalogramas (EEG) durante su estancia, el primero (dia 2 de hospitalización) con signos de encefalopatía grave y descargas epileptiformes interictales muy frecuentes en región fronto-central derecha con propagación a línea media y área homóloga contralateral; al día siguiente mostró aplanamiento difuso y nula reactividad a estímulos, patrón periódico a baja frecuencia y nula variabilidad en línea media. Finalmente, el último trazo electroencefalográfico (día 7 de hospitalización) presentó depresión difusa y permanente de la actividad eléctrica cerebral. Se evidenció ritmo eléctrico cerebral en frecuencia delta con nula variabilidad y ninguna respuesta a estímulos, pero sin cumplir criterios de silencio eléctrico cerebral.

Al quinto día desde el ingreso, se encontró ausencia de respuesta motora y de reflejos del tallo cerebral. De forma llamativa se describe la presencia de movimientos oculares verticales disociados (probable variedad de skew) que se exacerbaron durante la búsqueda del reflejo vestíbulo-ocular al estimular con agua fría. Presentaba ausencia de movimientos horizontales y respuesta vertical paradójica, sugiriendo compromiso extenso del tallo cerebral con predominio de daño protuberancial en núcleos vestibulares centrales con indemnidad del eje vertical. Posteriormente el protocolo de muerte encefálica se pospuso dado hipernatremia asociada, por lo que se solicitó un estudio ultrasonográfico por doppler transcraneal el día 7 de hospitalización, en el cual se evidenciaron signos de hipertensión endocraneana, caracterizados por aumento de las velocidades pico sistólicas y medias, así como dilatación de las vainas de los nervios ópticos, sin que se cumplieran criterios de parada circulatoria cerebral. Finalmente, a los 10 días de su ingreso se realizó un test de apnea de 8 minutos, con una pCO_2_ inicial de 46 mmHg y una pCO_2_ final de 80 mmHg, el cual resultó positivo, confirmándose el díagnóstico de muerte encefálica.

La necropsia reportó extensa destrucción del tejido encefálico con cuerpos de Negri en múltiples localizaciones, el diagnóstico de rabia humana se confirmó con inmunofluorescencia directa positiva en tejido cerebral. Las investigaciones en campo realizadas por instituciones gubernamentales informaron el contacto de la paciente con un felino silvestre (Tigrillo) que llegó a su vivienda con signos de enfermedad grave y que falleció 6 semanas antes del inicio de síntomas de la paciente. El linaje viral detectado fue de murciélago hematófago. La paciente no recibió inmunoglobulina antirrábica, pues nunca previo a su ingreso reportó mordeduras de animales o contacto con fauna silvestre.

## 3. Discusión

El caso corresponde a una encefalitis aguda rápidamente progresiva; se destacan en su presentación aparte de la alteración del estado mental, las crisis epilépticas, el compromiso disautonómico y la cuadriparesia flácida (siendo esta última la que desvió el diagnóstico certero del cuadro en su inicio). La exposición tanto a animales domésticos como fauna silvestre fue fundamental para sospechar rabia humana como etiología. La rabia humana es una zoonosis, causada por diferentes especies de virus neurotrópicos del género Lyssavirus de la familia Rhabdoviridae. Se estima que en el mundo ocurren 56.000 muertes por rabia humana, el 99% de ellas asociadas a caninos enfermos, en su mayoría en África y Asia (96%). En países donde existen programas de vacunación a animales domésticos y que tienen acceso a profilaxis postexposición (PEP) con inmunoglobulina, las muertes por rabia humana son infrecuentes y se asocian a fauna silvestre. En el continente americano, para el 2024 se reportaron tan sólo 22 muertes por rabia. En Colombia, no se reportaban casos desde el 2021, sin embargo, entre el año 2000 y el 2021 se confirmaron 89 muertes, la mayoría asociadas a exposición a gatos o murciélagos [1, 2].

Su presentación clínica típica corresponde a un espectro bifásico con una fase prodrómica inespecífica, seguida de la fase neurológica que puede ser encefalitis (la más frecuente) o paralítica. En su fase de encefalitis, los pacientes pueden presentar agitación, alucinaciones, hiperactividad simpática y crisis convulsivas; a diferencia de ésta, la fase paralítica progresa a cuadriparesia y coma [3, 4]. Entre los diagnósticos diferenciales se contempló el síndrome de Guillain-Barré (SGB) y encefalitis autoinmune. La fiebre persistente, el compromiso cortical temprano, las alucinaciones visuales, la ausencia de respuesta a inmunoglobulina intravenosa y la ausencia de disociacion inmunocitológica del LCR hicieron SGB improbable [5]. Con respecto a la encefalitis autoinmune, la evaluación diagnóstica se vio limitada por la imposibilidad de realizar una resonancia magnética cerebral secundaria a la inestabilidad hemodinámica de la paciente. No obstante, el panel inmunológico y los anticuerpos específicos fueron negativos, reduciendo entonces dicha posibilidad.

**Fig. 1.**
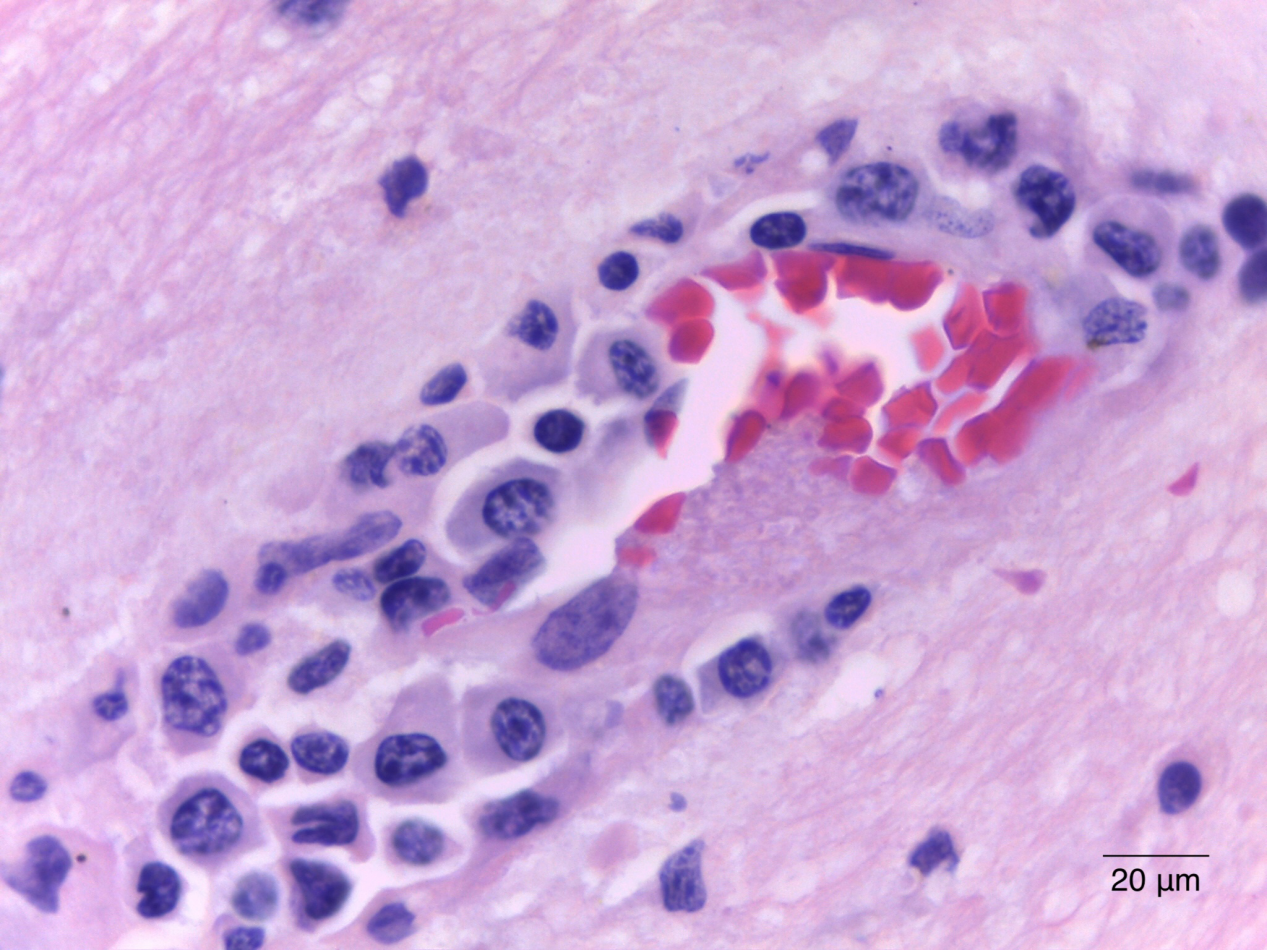
**Sustancia blanca del lóbulo frontal que muestra manguitos perivasculares prominentes, compuestos por infiltrado inflamatorio mononuclear**. (H&E 1000×). H&E, tinción con hematoxilina y eosina. Barra de escala = 20 μm.

**Fig. 2.**
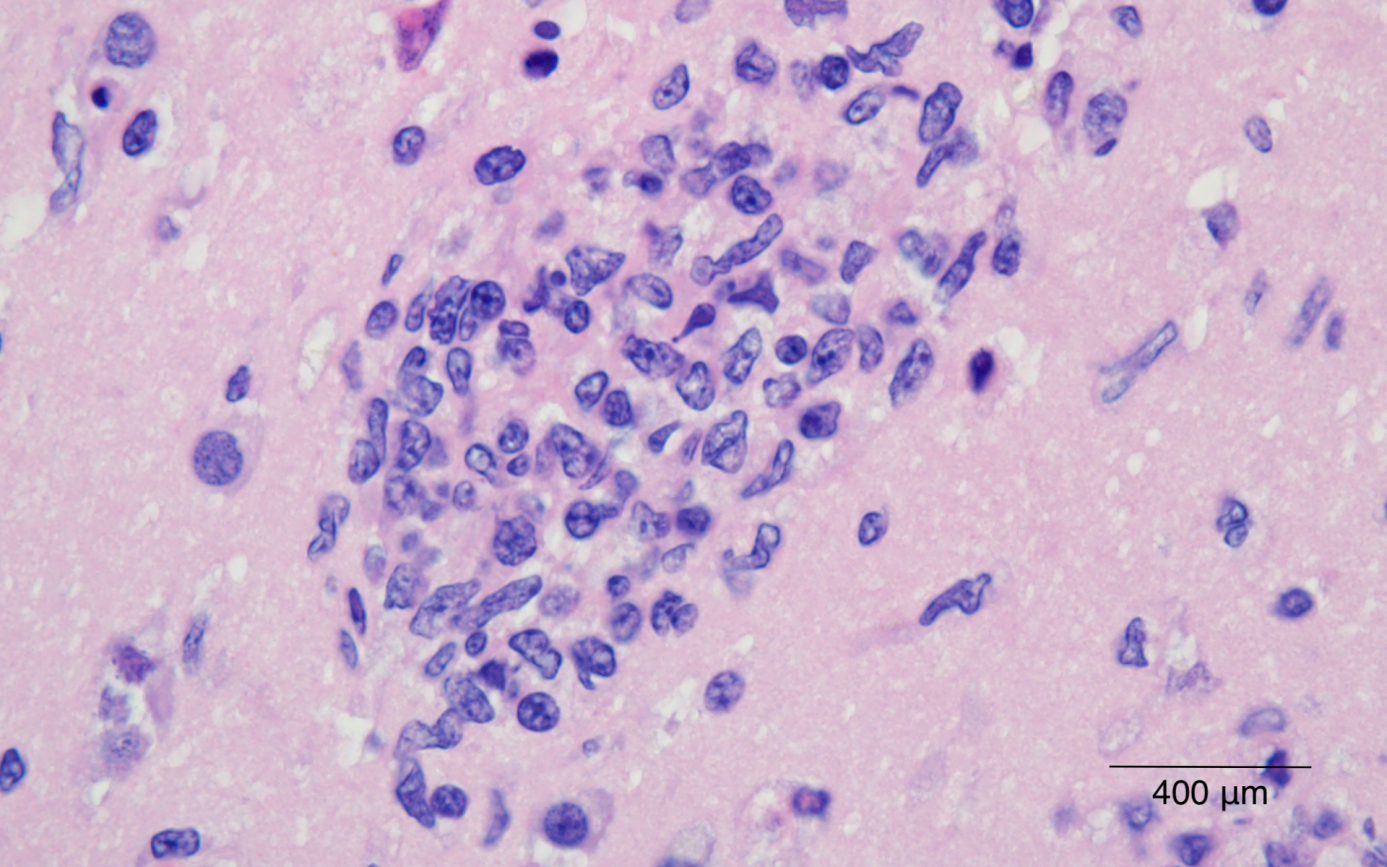
**Sustancia blanca del hipocampo con presencia de un nódulo microglial compuesto por células de núcleos alargados y pleomorfismo leve**. (H&E 400×). Barra de escala = 400 μm.

**Fig. 3.**
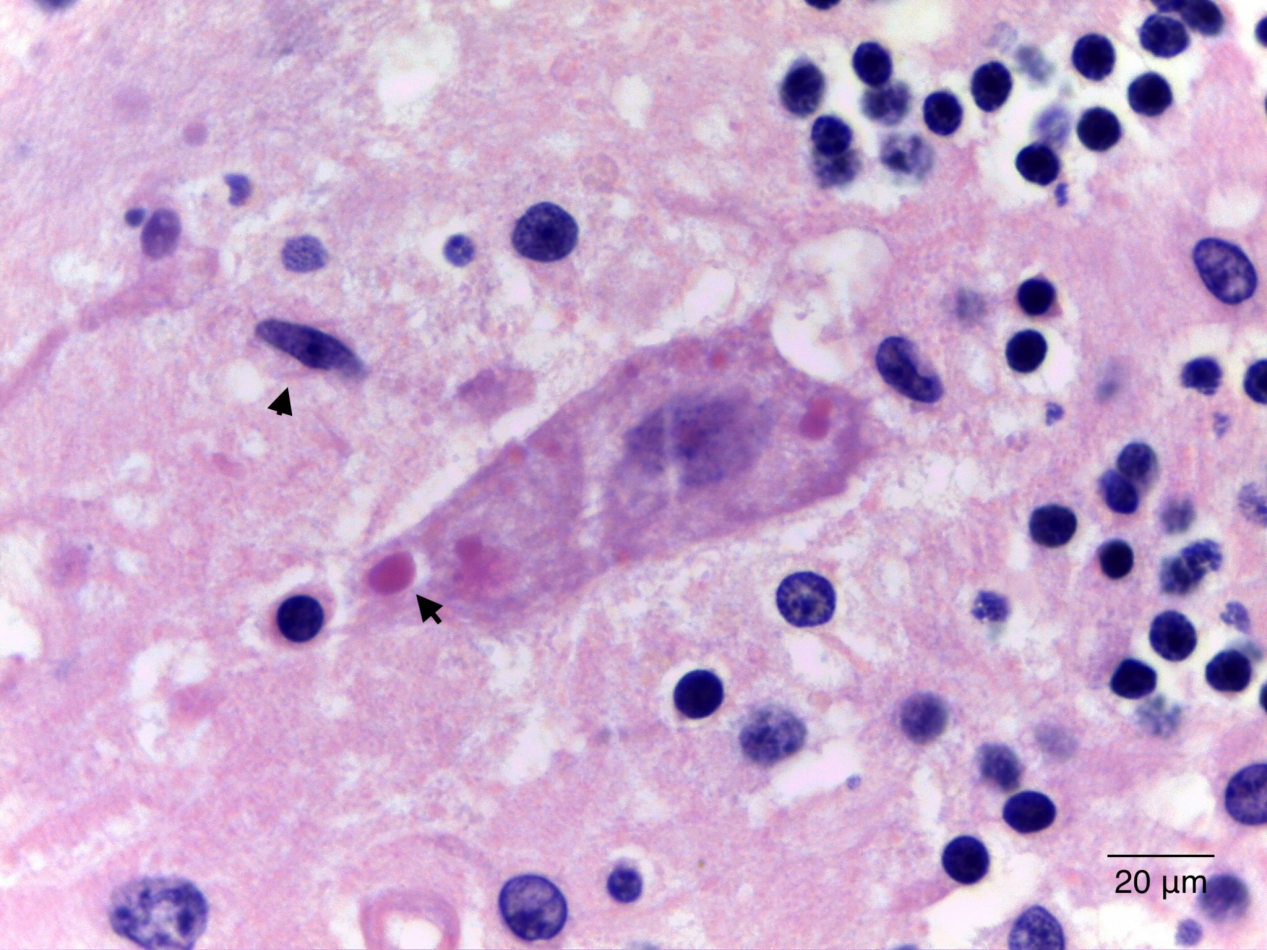
**Citoplasma de las células de Purkinje en el cerebelo con inclusiones eosinófilas redondeadas y bien definidas, de aproximadamente 7 µm de diámetro, compatibles con cuerpos de Negri (flecha) e inclusiones de menor tamaño correspondientes a cuerpos de Lyssa**. Además, se observan astrocitos reactivos (cabeza de flecha). (H&E 1000×). Barra de escala = 20 μm.

El diagnóstico de la rabia humana raras veces se hace antemortem, debido a su rápida progresión hacia la muerte y a la sensibilidad variable de las metodologías utilizadas, requiriendo para su diagnóstico múltiples muestras como biopsia de piel de la nuca, saliva, suero, o líquido cefalorraquídeo, utilizando múltiples técnicas como detección de anticuerpos, reacción en cadena de la polimerasa con transcripción inversa (RT-PCR) e inmunofluorescencia [6]. En Colombia el diagnóstico se hace posmortem usando inmunofluorescencia en el sistema nervioso central. En la histopatología cerebral suele evidenciarse inflamación perivascular, neuronofagia y cuerpos de Negri [7]. En este caso se documentaron los tres hallazgos mencionados (Figs. 1,2,3). Al examen microscópico se observó una extensa reacción perivascular mononuclear que afectaba también los vasos leptomeníngeos; numerosos nódulos microgliales dispersos alrededor de toda la sustancia blanca y adicionalmente neuronofagia que comprometía tanto la corteza como la sustancia gris subcortical. Las inclusiones citoplasmáticas eosinófilas, compatibles con los cuerpos de Negri, se observaron en cerebelo, hipocampo, hipotálamo, tallo y corteza cerebral.

Pese a los avances en la investigación médica, a la fecha no existe un tratamiento curativo efectivo una vez iniciados los síntomas, el manejo es paliativo [6, 8]. La importancia en su diagnóstico radica en que nuevos casos pueden prevenirse en la comunidad del paciente mediante diversas medidas de salud pública. Estas medidas incluyen: (1) Profilaxis postexposición con inmunoglobulina igG y vacuna antirrábica a todo contacto estrecho con el paciente o la fauna afectada; (2) Vacuna antirrábica anual a todos los mamíferos domésticos; (3) Educación a la comunidad para la consulta temprana y administración de PEP luego de todo accidente con un animal potencialmente transmisor de rabia [6, 9, 10].

## 4. Conclusiones

La rabia humana, aunque infrecuente en el continente americano [1], continúa siendo una amenaza mortal, especialmente en áreas rurales donde el contacto con animales domésticos y silvestres es frecuente. Su variante paralítica, puede confundirse inicialmente con Síndrome de Guillain Barré, pero la presencia de encefalitis asociada, la parálisis asimétrica, la disautonomía y la ausencia de disociación inmunocitología del líquido cefalorraquídeo, deben generar la sospecha de rabia humana. El diagnóstico es fundamental para prevenir otras muertes, mediante el acceso a la profilaxis postexposición luego de la mordedura por un mamífero potencialmente transmisor de rabia, la vacunación universal de animales domésticos y la educación a la comunidad sobre los riesgos de manipular fauna silvestre [6].

## Data Availability

Los datos que respaldan los hallazgos de este estudio no están disponibles públicamente debido a consideraciones de confidencialidad, pero pueden ser solicitados al autor correspondiente bajo criterios razonables.

## Contribuciones de los Autores

FAGJ, PAVR, KTO y SML contribuyeron al diseño del estudio, la recolección y análisis de los datos, así como a la redacción y revisión crítica del manuscrito. JCAV y JDCR realizaron la necropsia epidemiológica y toma de microfotografías. Todos los autores contribuyeron a la revisión editorial del manuscrito. Todos los autores aprobaron la versión final del manuscrito. Todos los autores han participado suficientemente en el trabajo y están de acuerdo en asumir la responsabilidad de todos los aspectos del mismo.

## Aprobación Ética y Consentimiento Informado

El estudio se realizó de acuerdo con los principios de la Declaración de Helsinki y fue aprobado por el Comité de Ética en la Investigación de la Fundación Hospitalaria San Vicente de Paul (Medellín, Antioquia) el día 28 de noviembre de 2025 según consta en el Acta N° 32-2025 con exención del consentimiento informado dado que la paciente falleció durante la atención y no fue posible localizar a los familiares posteriormente. Esto teniendo en cuenta que no se exponen datos sensibles y que se controla la información para evitar que se reconozca la identidad de la paciente.

## Financiación

Esta investigación fue financiada en su totalidad por la Fundación Hospitalaria San Vicente de Paul (Medellín, Antioquia).

## Material Suplementario

El material suplementario asociado con este artículo se puede encontrar, en la versión en línea, en https://doi.org/10.31083/RN49292.
